# Case Report: Persistent urogenital sinus (PUGS) associated with complete bicorporeal uterus, double cervix and partial longitudinal non-obstructing vaginal septum: a one-stop diagnostic and operative hysteroscopic approach

**DOI:** 10.3389/fsurg.2026.1890509

**Published:** 2026-08-13

**Authors:** Emma Bonetti Palermo, Michela Zorzi, Federica Pozzati, Federico Ferrari, Franco Odicino, Antonia Carla Testa, Ursula Catena

**Affiliations:** 1 S.C. Ostetricia e Ginecologia, ASST Spedali Civili di Brescia, Dipartimento Area della Donna e Materno Infantile, Brescia, Italy; 2 Ovarian Cancer Center, Candiolo Cancer Institute, FPO-IRCCS, Torino, Italy; 3 Dipartimento Scienze Della Salute Della Donna e del Bambino, Fondazione Policlinico Universitario A. Gemelli, IRCCS, Rome, Italy; 4 Department of Clinical and Experimental Sciences, University of Brescia, ASST Spedali Civili, Brescia, Italy; 5 G-AURIG–Gemelli Advanced Ultrasound Research and Innovation Group, Fondazione Policlinico Universitario Agostino Gemelli, IRCCS, Rome, Italy; 6 Gemelli Women’s Health Center for Digital and Personalized Medicine, Dipartimento Scienze Della Vita e Sanità Pubblica, Università Cattolica del Sacro Cuore, Rome, Italy

**Keywords:** complete bicorporeal uterus, double cervix, hysteroscopy, müllerian malformations, persistent urogenital sinus (PUGS), vaginal septum

## Abstract

**Introduction:**

Persistent urogenital sinus (PUGS) is a rare congenital malformation characterized by the confluence of the urethra and vagina into a single distal channel. It may be associated with other congenital anomalies, including Müllerian malformations. Although typically diagnosed in childhood, delayed diagnosis may occur, particularly in patients with mild or nonspecific symptoms.

**Case presentation:**

We report the case of a 39-year-old nulliparous woman referred for suspected complex genital malformation, presenting with chronic pelvic pain, dyspareunia, and recurrent urogenital infections. A one-stop diagnostic and operative approach, combining transvaginal ultrasound and hysteroscopy, identified PUGS associated with a complete bicorporeal uterus, double cervix, and partial longitudinal non-obstructing vaginal septum (U3bC2V1 according to the ESHRE/ESGE classification). A 15-Fr bipolar mini-resectoscope was used for incision of the vaginal septum and removal of a concomitant FIGO type 0 fibroid in the right hemiuterus. The procedure was completed without complications.

**Discussion:**

The integration of expert ultrasound and endoscopic evaluation enabled accurate anatomical characterization and guided tailored surgical management. The use of a 15-Fr bipolar mini-resectoscope allowed effective treatment in a complex anatomical setting, avoiding cervical dilation while providing adequate maneuverability and simultaneous cutting and coagulation.

**Conclusion:**

A one-stop approach in a specialized center may improve diagnostic accuracy and support safe, individualized minimally invasive management of complex congenital urogenital anomalies.

## Introduction

Persistent urogenital sinus (PUGS) is a rare congenital malformation, with an incidence of approximately 6 per 100,000 females, defined by the confluence of the urethra and vagina into a single distal channel opening through a common perineal orifice (1, 2).

Although the urogenital sinus is a normal transient structure during embryologic development, its persistence may result from abnormal embryogenesis or occur in the context of disorders of sex development (DSD), among which congenital adrenal hyperplasia (CAH) is the most common, accounting for approximately 80% of all DSD cases (1).

CAH represents the main endocrine condition associated with PUGS. It leads to 21-hydroxylase deficiency with a consequent androgen excess, resulting in variable degrees of virilization, including clitoral hypertrophy, and the severity of external genital virilization may correlate with the length of the common urogenital channel (1, 3).

PUGS may also be associated, in approximately 33% of cases, with other congenital anomalies, including renal, anorectal, and Müllerian malformations (4). While Müllerian anomalies are often diagnosed during the evaluation of infertility, recurrent pregnancy loss, or pelvic symptoms, PUGS is usually identified in childhood due to urinary or genital symptoms. However, delayed diagnosis may occur, particularly in pauci-symptomatic patients or in cases with subtle external genital findings (5).

Accurate anatomical assessment is essential for diagnosis and surgical planning. The combination of two- and three-dimensional ultrasound and endoscopic evaluation may provide a comprehensive, minimally invasive diagnostic approach (4).

We report the case of an adult patient diagnosed with PUGS associated with a complex Müllerian malformation, including complete bicorporeal uterus, double cervix, and partial longitudinal non-obstructing vaginal septum. The diagnosis and treatment were achieved through a one-stop approach integrating transvaginal ultrasound with diagnostic and operative hysteroscopy in a single session.

## Case description

### Patient information and clinical presentation

A 39-year-old nulliparous woman was referred to the Gynecological Department of Fondazione Policlinico Universitario Agostino Gemelli IRCCS, Rome, Italy, for evaluation of a suspected complex genital malformation. She reported regular menstrual cycles, dysmenorrhea, dyspareunia, chronic pelvic pain, and recurrent urogenital tract infections. She presented with a desire for pregnancy and a 4-year history of infertility. Her past medical history was unremarkable, and no family history of congenital urogenital anomalies was reported.

The patient was scheduled for a one-stop diagnostic and operative procedure, combining transvaginal ultrasound and hysteroscopy, at the Digital Hysteroscopic Clinic (DHC)—CLASS Hysteroscopy, under general anaesthesia (laryngeal mask), according to an ambulatory model of care (6, 7).

### Diagnostic assessment

**Figure 1:**
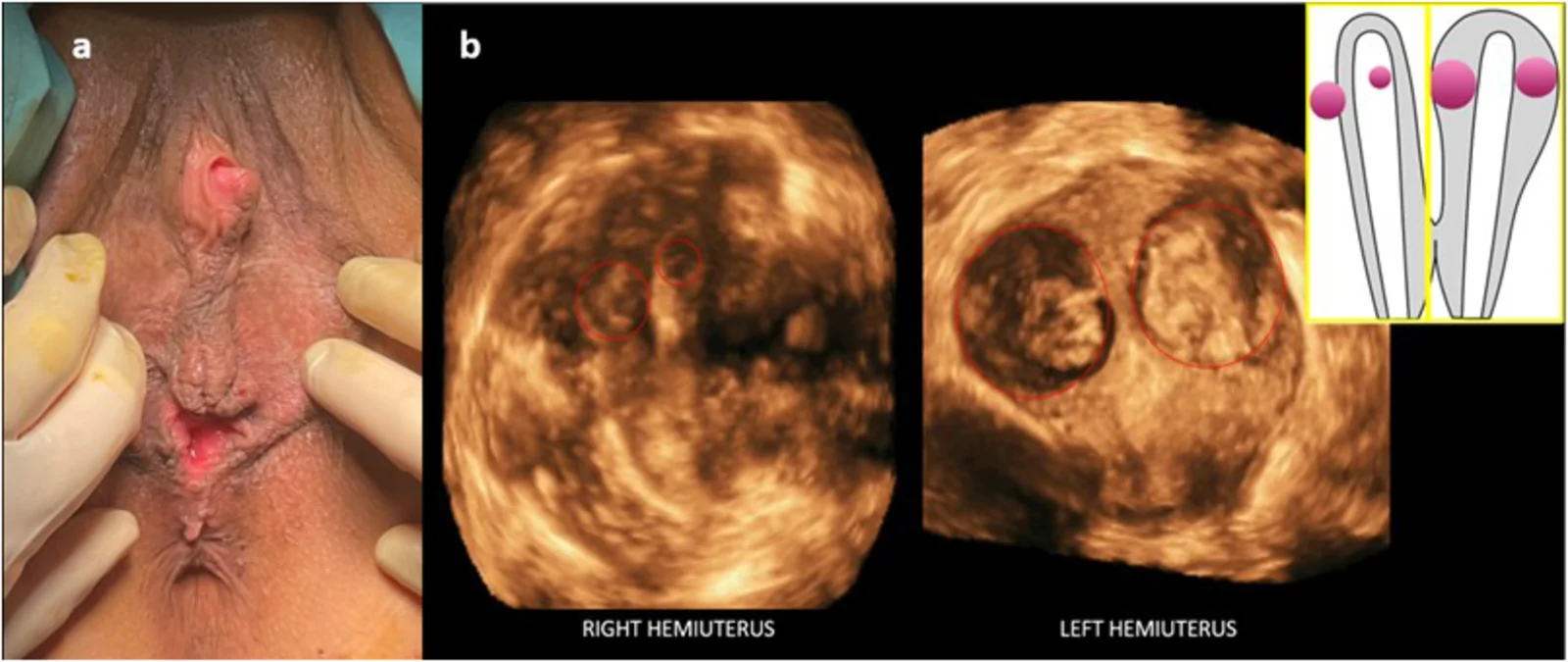
Preoperative assessment: **(a)** external genitalia showing clitoral hypertrophy and partial midline fusion of the labia minora; **(b)** three-dimensional transvaginal ultrasound reconstruction showing a complete bicorporeal uterus, with the right hemiuterus containing a FIGO type 0 myoma and an intramural-subserosal myoma, and the left hemiuterus distorted by two intramural-subserosal myomas.

External genital examination revealed clitoral hypertrophy and partial midline fusion of the labia minora; the urethral meatus was not initially identifiable (Figure 1a).

Two-dimensional transvaginal ultrasound with three-dimensional (3D) reconstruction revealed a complete bicorporeal uterus; a 10-mm FIGO type 0 fibroid was found in the right hemiuterus, with an additional intramural-subserosal fibroid, while the left hemiuterus was distorted by two intramural-subserosal fibroids (Figure 1b). No associated renal anomalies were identified. No additional imaging, including magnetic resonance imaging (MRI), was required.

**Figure 2:**
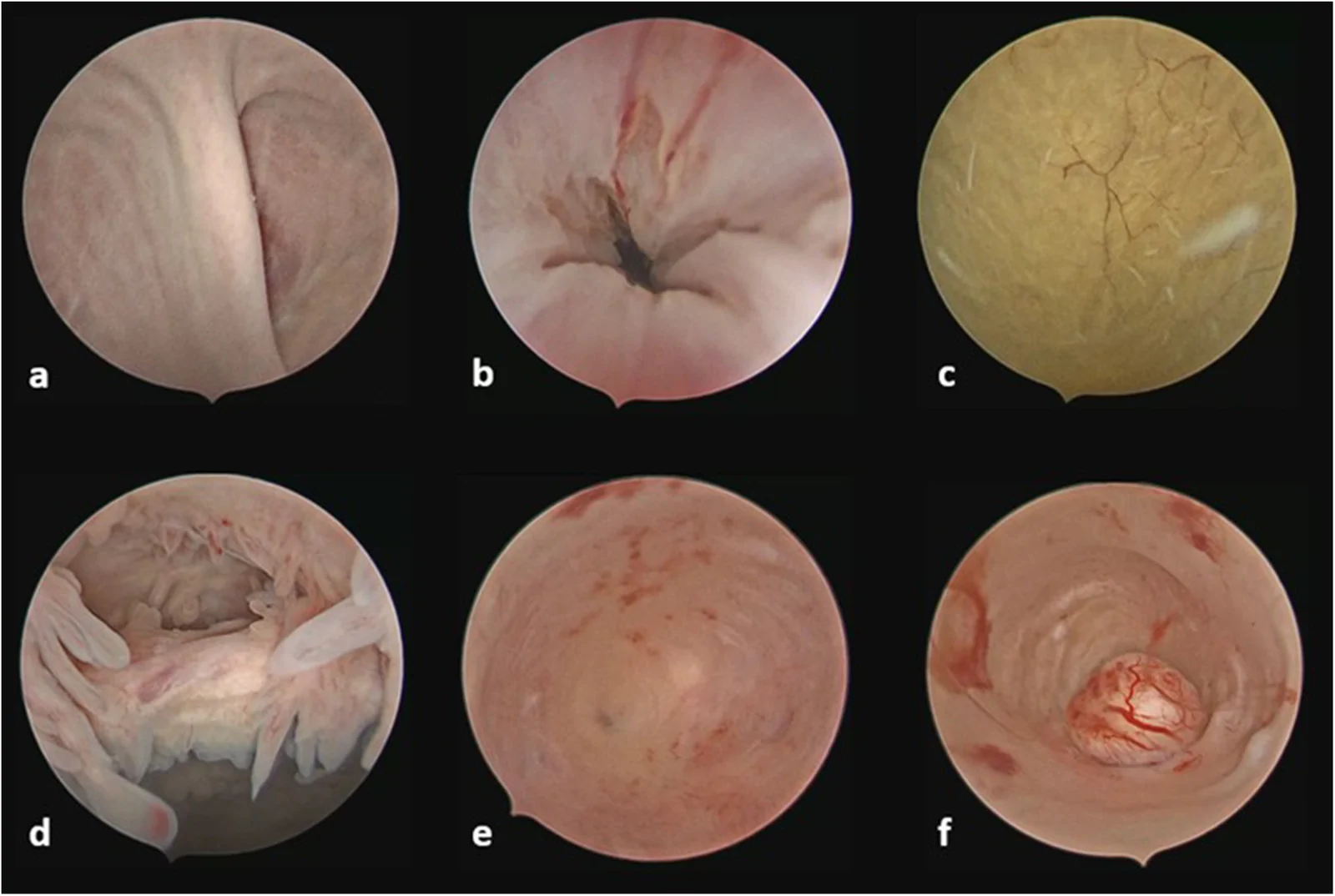
Diagnostic hysteroscopy: **(a)** partial longitudinal non-obstructing vaginal septum; **(b)** identification of the urethral lumen during withdrawal of the hysteroscope; **(c)** bladder access through the urethral lumen; **(d)** single distal channel consistent with PUGS; **(e)** regular left uterine cavity; **(f)** right uterine cavity with a FIGO type 0 myoma located on the posterior wall.

Diagnostic hysteroscopy was performed using a 5-mm Bettocchi hysteroscope (Karl Storz, Tuttlingen, Germany) with a vaginoscopic approach (Supplementary Video S1). Initial endoscopic evaluation of the vaginal canal revealed a partial longitudinal non-obstructing vaginal septum and a double cervix (Figure 2a). During gradual withdrawal of the instrument while maintaining a ventral orientation, the urethral lumen was identified (Figure 2b), allowing access to the bladder (Figure 2c). Further endoscopic visualization during withdrawal demonstrated a single distal common urogenital channel, approximately 10 mm in length from the external perineal orifice to the bifurcation, consistent with PUGS, bifurcating anteriorly into the urethra and posteriorly into the vagina (Figure 2d).

Both uterine hemi-cavities were explored hysteroscopically through their respective cervical canals: the left cavity appeared regular (Figure 2e), whereas the right cavity showed the 10-mm FIGO type 0 myoma located on the posterior wall (Figure 2f).

The combined imaging and endoscopic findings established the diagnosis of PUGS associated with a complete bicorporeal uterus, double cervix, and partial longitudinal non-obstructing vaginal septum (U3bC2V1 according to the ESHRE/ESGE classification system) (8).

### Operative hysteroscopy

**Figure 3:**
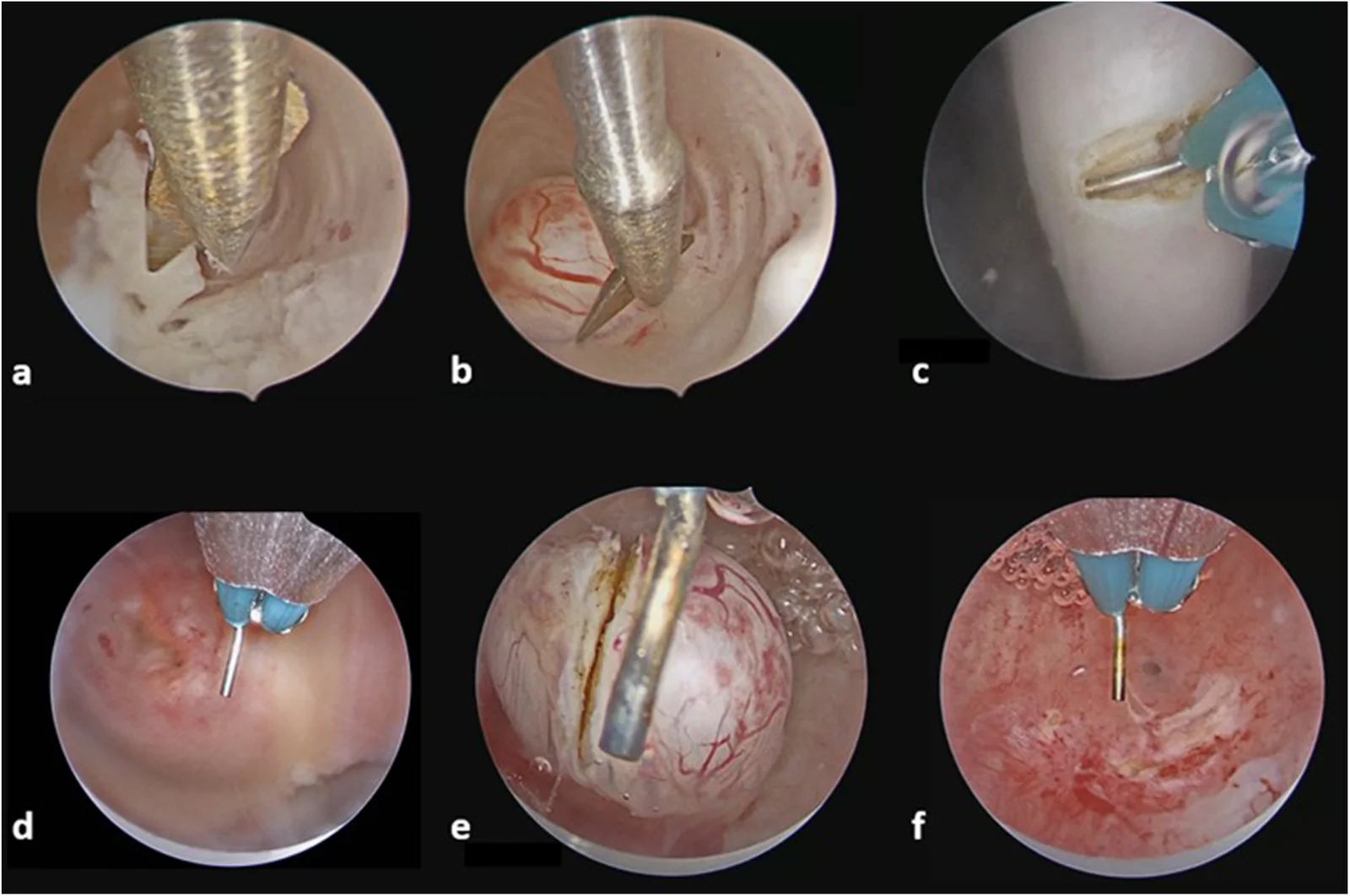
Operative hysteroscopy: **(a)** endometrial biopsy of the uterine hemi-cavities using 5-Fr instruments; **(b)** incision of the base of the FIGO type 0 myoma in the right uterine cavity; **(c)** caudo-cranial incision of the vaginal septum using a 15-Fr bipolar mini-resectoscope with a Collins loop; **(d)** complete opening of the vaginal septum up to the level of the two cervices; **(e)** division of the myoma into two fragments; **(f)** complete resection of the submucosal myoma.

The operative hysteroscopy was subsequently performed (Supplementary Video S1). Endometrial biopsies of both the left and right uterine cavities were obtained using 5-Fr miniaturized cold instruments through a 5-mm hysteroscope (Figure 3a). Partial myomectomy of the submucosal fibroid in the right uterine cavity was then performed using 5-Fr scissors by incising the base of the lesion (Figure 3b).

A 15-Fr bipolar mini-resectoscope (Karl Storz, Tuttlingen, Germany) with a Collins loop was subsequently used to incise the vaginal septum in a caudo-cranial direction (Figure 3c). The septal incision was extended cranially up to the level of the two cervices, achieving complete opening of the vaginal septum (Figure 3d).

Using the same instrument, the myomectomy was then completed by dividing the fibroid into two fragments (Figure 3e). This facilitated removal through the right cervical canal and allowed complete resection of the submucosal component (Figure 3f).

The procedure was completed in 11 min without intraoperative complications. The patient was discharged 3 h after surgery. No postoperative complications occurred.

At the 9-month postoperative follow-up, the patient reported resolution of dyspareunia after resection of the longitudinal vaginal septum. Given the relatively short follow-up period, no definitive reproductive outcomes can yet be reported.

## Discussion

**Figure 4:**
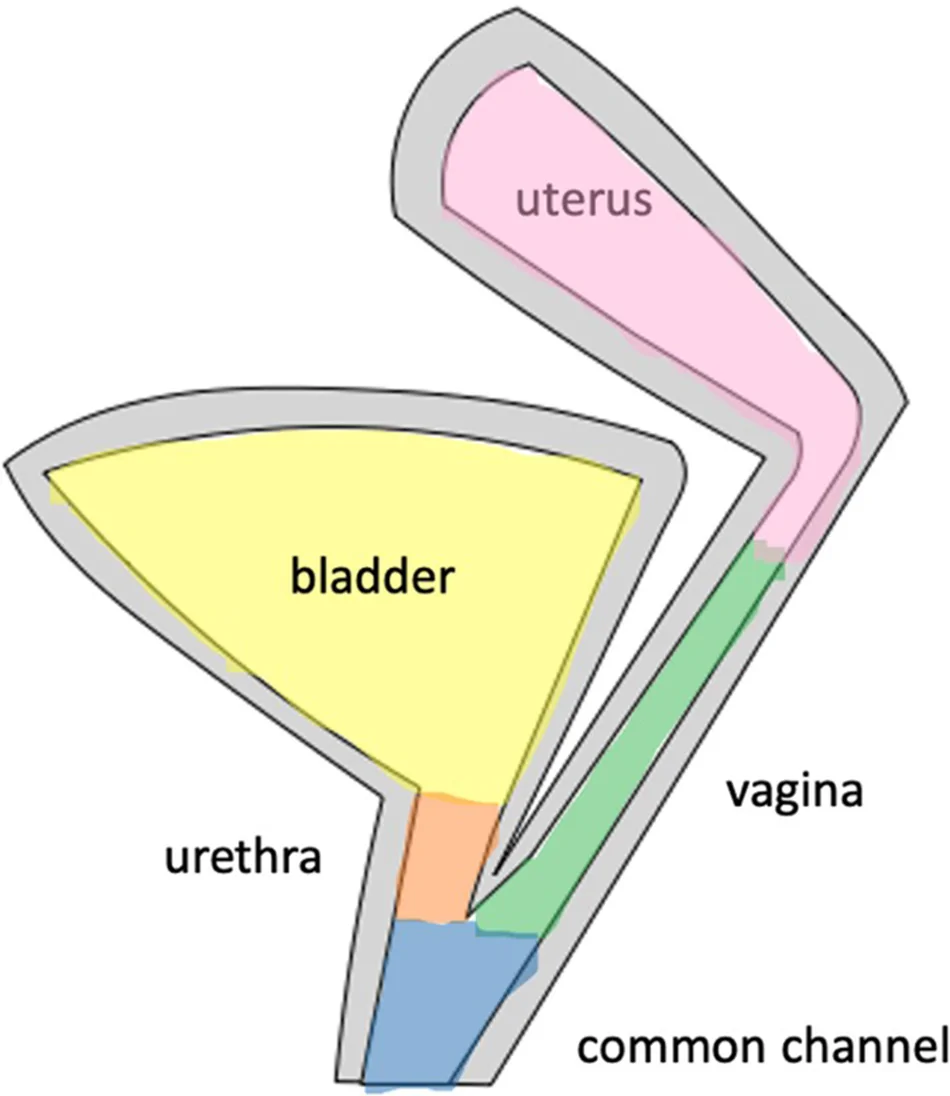
Schematic representation of persistent urogenital sinus (PUGS).

Persistent urogenital sinus (PUGS) is a rare condition characterized by an abnormal confluence of the urethra and vagina into a single distal channel (1, 2). This channel is typically wider and longer than a normal urethra and is lined by squamous epithelium, similar to vaginal mucosa. Anatomically, the vagina usually opens into the posterior or posterolateral wall of the sinus, while the urethral tract continues cranially toward the bladder, resulting in a complex configuration, schematically represented in Figure 4.

Due to its rarity and the limited awareness, PUGS may remain undiagnosed until adulthood, particularly in patients with mild obstructive symptoms or nonspecific complaints. In our case, the condition remained unrecognized until adulthood, despite the presence of chronic pelvic symptoms and recurrent urogenital infections, highlighting the risk of underdiagnosis and its potential impact on quality of life.

PUGS may coexist with other congenital anomalies, including Müllerian malformations, in approximately one-third of cases (4). In the present case, PUGS was associated with a complete bicorporeal uterus, double cervix, and partial longitudinal non-obstructing vaginal septum (U3bC2V1 according to the ESHRE/ESGE classification system) (8).

From a therapeutic perspective, surgical management was tailored to address the patient's symptoms. The partial longitudinal vaginal septum was resected, as it may contribute to dyspareunia and pelvic discomfort; notably, the patient reported resolution of dyspareunia at the 9-month postoperative follow-up, supporting the clinical relevance of septum resection in this case. Conversely, the complete bicorporeal uterus and double cervix were left *in situ*, as these anatomical variants do not require surgical correction in the absence of a specific indication (9).

The same surgical procedure also allowed hysteroscopic removal of the 10-mm FIGO type 0 myoma in the right hemiuterus. Considering the patient's infertility history and her desire for pregnancy, removal of the FIGO type 0 myoma may additionally optimize the right uterine cavity for future implantation. However, longer follow-up is needed to assess reproductive outcomes.

A 15-Fr bipolar mini-resectoscope was used for both septum incision and myoma resection. This instrument offers several advantages in complex genital anatomy: it does not require cervical dilation, provides excellent maneuverability in narrow or anatomically distorted spaces, and allows simultaneous cutting and coagulation (10). In the present case, the use of the mini-resectoscope also enabled division of the 10-mm submucosal myoma into two fragments, facilitating removal through the right cervical canal and allowing complete resection of the submucosal component. These features are particularly relevant in patients with congenital malformations, in whom minimizing trauma and maintaining precise endoscopic control are essential. Furthermore, the vaginoscopic approach to vaginal septum incision offers significant advantages over the traditional vaginal technique. It avoids the use of speculum and traction instruments, reduces bleeding and tissue trauma, and provides superior visualization of the lower genital tract (11).

Although the external genital appearance suggested a narrow introitus, intraoperative assessment confirmed that the introitus was clinically adequate, and no labial adhesiolysis or additional vulvar reconstructive surgery was therefore performed. While partial labial fusion may contribute to dyspareunia in selected cases, the resolution of dyspareunia reported after resection of the longitudinal vaginal septum supported the septum as the main surgically relevant contributor to her sexual symptoms in this case.

This case emphasizes the importance of referral to specialized centers with expertise in congenital genital tract anomalies. Accurate diagnosis and appropriate individualized management require a detailed anatomical assessment, which can be challenging when multiple malformations coexist (12). In this context, the integration of expert ultrasound and endoscopic evaluation represents a key diagnostic strategy. As reported by Mirandola et al., this integrated approach allows a dynamic assessment of complex urogenital anatomy, improving diagnostic accuracy while reducing the risk of misinterpretation and unnecessary invasive procedures. In the present case, the one-stop approach combining transvaginal ultrasound and diagnostic hysteroscopy performed by expert operators was crucial for both accurate diagnosis and optimal treatment planning.

## Conclusions

This case highlights the importance of accurate anatomical assessment in patients with complex congenital urogenital anomalies. The integration of expert ultrasound and endoscopic evaluation provides an effective one-stop diagnostic and operative minimally invasive management. Referral to specialized centers is essential to ensure optimal diagnosis and treatment.

## Data Availability

The original contributions presented in the study are included in the article/Supplementary Material, further inquiries can be directed to the corresponding author.
